# Modified minimally invasive surgery in reconstructing the temporomandibular joint disk by transplantation of the temporalis myofascial flap

**DOI:** 10.1186/s12891-023-06128-z

**Published:** 2023-01-05

**Authors:** Chen-xi Li, Peng Yu, Zhong-cheng Gong, Xu Liu

**Affiliations:** 1 Department of Oral and Maxillofacial Oncology & Surgery, The First Affiliated Hospital of Xinjiang Medical University, School/Hospital of Stomatology Xinjiang Medical University, Stomatological Research Institute of Xinjiang Uygur Autonomous Region, No.137 Liyushan South Road, Urumqi, 830054 People’s Republic of China; 2 Department of Maxillofacial Surgery, Hospital of Stomatology, Key Laboratory of Dental-Maxillofacial Reconstruction and Biological Intelligence Manufacturing of Gansu Province, Faculty of Dentistry, Lanzhou University, Lanzhou, 730013 People’s Republic of China

**Keywords:** Temporomandibular joint disorders, Pedicled temporalis myofascial flap, Microsurgical repair and reconstruction

## Abstract

**Objective:**

The transplantation of temporalis myofascial flap (TMF) is an indispensable method to treat severe temporomandibular joint disorders with disk failure. How to select the indication and the ways of transplantation is still a challenging topic for achieving the ideal effect. We reported the new methods and follow-up results of the patients treated with pedicled TMF transplantation.

**Methods:**

Retrospective case series was performed at Temporomandibular Joint Specialist Clinic, the First Affiliated Hospital of Xinjiang Medical University, from December 2014 to August 2022. 39 patients (50 sides) included anterior disk displacement without reduction and disk perforation who underwent discectomy and immediate reconstruction with pedicled TMF. The initial and postoperative maximum mouth opening (MMO), and pain visual analogue scale (VAS) were compared via paired *t*-test and Wilcoxon signed-rank sum test, respectively.

**Results:**

The average of follow-up time was 30.07 months. The reconstructed temporomandibular joints basically achieved stable occlusion, good function, and satisfactory effect. The patients displayed a remarkable reduction of VAS score of pain, and improvement of the MMO compared with that before operation (*P* <  0.001). MR scanning revealed the grafts in joint space showed band-shaped soft tissue density of medium signal and had the clear edges, without interruption and fragment.

**Conclusion:**

Reconstruction of the temporomandibular joint disk by transplantation of the TMF applying modified minimally invasive surgery was a feasible method, which could improve the function of joints and prevent adhesion, without obvious complications in donor region.

## Introduction

Temporomandibular joint disorders (TMD) is a set of conditions affecting the jaw joints and surrounding muscles and ligaments, which can be caused by psychological factors, an improper bite, bruxism (teeth grinding/ clenching), acute trauma, arthritis or wear and tear [1, 2]. There is also evidence that TMD may be related to cervical spine disorders and its mobility [2]. Although sleeping problems have been hypothesized as an etiological or risk cofactor, the degree to which it plays a role has not been definitively delineated [3, 4]. The prevalence of TMD ranges from 15 to 54% in different populations, and most affected individuals can present with characteristic symptoms such as clicking, joint pain, limited range of mouth opening, masticatory difficulty, mandible dysfunction, *etc* that the peak of the development of the symptoms is between 20 and 40 years of age [5, 6]. Recurrent attacks of severe chronic TMD many times may result in the degeneration/ derangement of articular disk, so as to further lead to functional morbidity like compromised speech, mastication, and deglutition, and even unaesthetic outcome in poor situation [7–9].

The temporomandibular joint (TMJ) disk (also called meniscus) is comprised of fibrocartilage, with crimped collagen, reckoned to better absorb impacts. On the one hand, the disk itself locates between the condyle of the jaw and mandibular fossa, merely comprising coarse nonvascular connective cells; therefore, disk displacement/peroration will occur when the biochemical and biomechanical loads tremendously exceed the normal levels that the temporomandibular disk can withstand [10–12]. On the other hand, it is characterized by a biconcave shape with a thicker periphery attached to the TMJ capsule and divides the joint into two compartments [8, 12]. Hence reconstruction of such sophisticated structure after ablative surgery is more challenging. Various loco-regional and free flaps and kinds of artificial biomaterials have been reported in extant literature nonetheless still an ideal reconstruction option for such structure is under research.

Except that no need to consider its biocompatibility, temporalis myofascial flap (TMF) seems to be one of the optimal choices among all other regional flaps since it is a locally available, reliable and safe flap characterized with abundant blood supply, adequate bulk and its anatomical site is near to damaged disk [13]. The purpose of this study was to investigate the efficacy of the TMF as an interpositional component transplanted for the reconstruction of TMJ disk. Many previous investigators have examined the use of TMF in TMJs that had assorted forms of existing pathological conditions such as congenital anomalies, autoimmune arthritides, degenerative joint disease, traumatic defects, lateral capsule flaccidity, ankyloses, as well as in those with prior alloplastic implants and autogenous grafts [14]. In this investigation, the TMFs were studied exclusively in patients whose joints had formerly been treated with conservative therapy and without any surgeries. This present study analyzed both preoperative and postoperative subjective and objective findings in TMD patients.

## Patients and methods

**Fig. 1:**
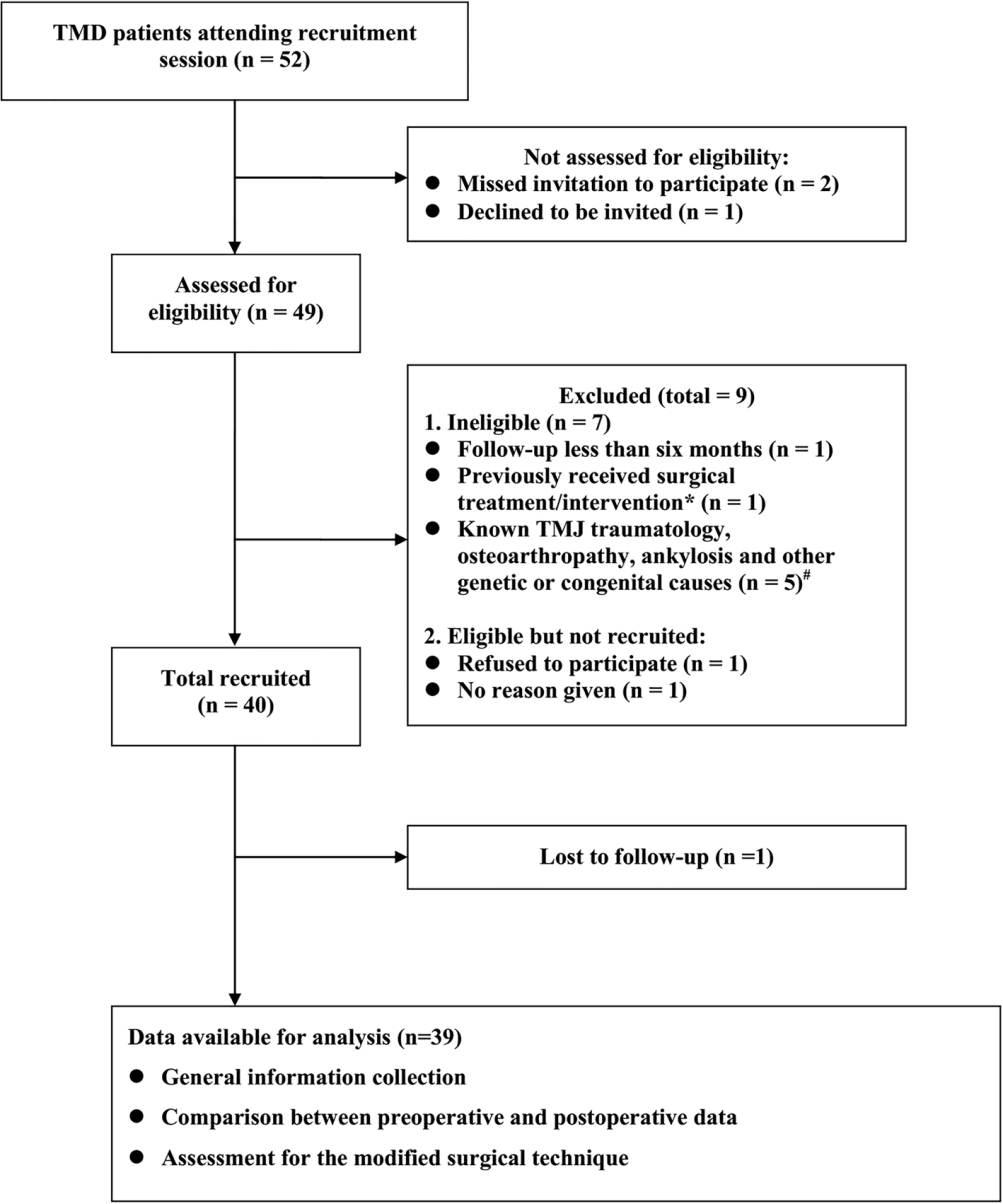
Study flowchart. *One patient had an operation history using artificial arthroplastic materials. ^#^Two patients suffered with condylar fracture, one suffered with subcortical cyst of condyle, one suffered with joint synovial chondromatosis, one suffered with TMJ ankyloses, respectively. Finally, all enrolled 39 patients were diagnosed as anterior disk displacement without reduction or disk perforation and accepted and underwent the modified minimally invasive surgery in reconstructing the temporomandibular joint disk by transplantation of the pedicled temporalis myofascial flap

At first, fifty-two consecutive TMD patients from December 2014 to August 2022 constituted the population of this study. By accessing medical record files, in total, thirty-nine patients composed of 5 males and 34 females were selected for the present study. Among them, 28 patients underwent unilateral TMF procedures and 11 patients underwent bilateral TMF procedures (Table 1). A flow diagram describing the subjects’ enrollment as well as the next working plan is given in Fig. 1.

**Table 1:** Included patients population demographics, and baseline data

Case no.	Gender (F/M)	Age (yr)	Affected side (L/R/Bi)	Previous treatment	Preoperative radiographic findings^a^		Diagnosis	Duration of disease course (mon)
					Disk deformities	Bony change		
**1**	F	42	Bi	Arthroplasty/proplast	Folded	Degeneration	OA, disk perforation	24
**2**	F	52	L	Sodium hyaluronate injection	Biconcave	Degeneration	OA	24
**3**	F	28	Bi	18 tooth extraction; electrothermal therapy	Biplanar	Condylar head displacement	OA	24
**4**	F	59	R	Self-administered prescription of oral antibiotics	Biconcave	Abnormal condylar morphology	OA, disk perforation	3
**5**	F	54	R	NM	Hemiconvexand	Degeneration	Nonreducing ADD, synovitis	12
**6**	M	60	L	Sodium hyaluronate injection	Biconvex	Degeneration	Nonreducing ADD, masseter and LPM edema	4
**7**	F	49	L	NM	Folded	Degeneration	OA	20
**8**	F	56	L	Sodium hyaluronate injection	Folded	Degeneration	OA, disk perforation	24
**9**	F	25	Bi	Sodium hyaluronate injection; stable occlusal splint	Biplanar	Abnormal condylar morphology	OA	5
**10**	F	28	R	Local anesthesia; stable occlusal splint	Folded	Abnormal condylar morphology	Nonreducing ADD	8
**11**	F	59	R	Required surgical treatment directly	Biconcave	Degeneration	OA	0.5
**12**	F	45	Bi	NM	Biconvex	Degeneration	Nonreducing ADD	3
**13**	F	30	Bi	NM	Biconvex	Degeneration	Nonreducing ADD	3
**14**	F	29	Bi	NM	Folded	Degeneration	OA, disk perforation	11
**15**	F	47	R	Hyaluronic acid injection		Degeneration	Nonreducing ADD	18
**16**	M	39	R	Hyaluronic acid injection	Biconvex	Degeneration	Nonreducing ADD	3
**17**	F	55	R	NM		Degeneration	Nonreducing ADD	14
**18**	M	62	R	NM	Atypical deformation	Degeneration	OA, disk perforation	NA
**19**	F	23	Bi	48 tooth extraction; hyaluronic acid injection; stable occlusal splint	Folded	Degeneration	OA, disk perforation	48
**20**	F	47	L	NM		Degeneration	OA, disk perforation	2
**21**	F	59	L	NM	Folded	Degeneration	OA, disk perforation	5
**22**	F	67	R	Local anesthesia	Folded	Degeneration	Nonreducing ADD	11
**23**	F	45	Bi	NM		Degeneration	OA, disk perforation	6
**24**	F	53	L	Traditional Chinese medicine therapy including acupuncture, cupping therapy, Tuina manual therapy, and herbal medicine	Biconcave	Degeneration	Nonreducing ADD	6
**25**	F	33	R	NM	Biconvex	Degeneration	OA, disk perforation	6
**26**	F	48	Bi	NM	Biconvex	Degeneration	Nonreducing ADD	12
**27**	F	48	Bi	NM	Biconvex	Degeneration	OA, disk perforation	10
**28**	F	35	L	NM	Biconvex	Degeneration	Nonreducing ADD	8
**29**	F	55	R	NM	Hemiconvexand	Degeneration	OA, disk perforation	24
**30**	F	51	L	Local anesthesia; sodium hyaluronate injection	Hemiconvexand	Degeneration	OA, disk perforation	2
**31**	F	30	R	NM	Biconcave	Degeneration	Nonreducing ADD	5
**32**	F	33	R	18 and 48 tooth extraction; hyaluronic acid injection	Folded	Degeneration	OA, disk perforation	NM
**33**	F	29	R	NM	Biconcave	Degeneration	Nonreducing ADD	12
**34**	M	57	L	Hyaluronic acid injection	Folded	Degeneration	OA, disk perforation	1
**35**	F	58	L	NM	Disk calcification	Degeneration	OA, disk perforation	1
**36**	F	42	L	Oral Meloxicam	Folded	Degeneration	OA, disk perforation	1
**37**	F	26	L	NM	Folded	Degeneration	OA, disk perforation	1.5
**38**	F	52	Bi	NM	Hemiconvexand	Degeneration	OA, disk perforation	1
**39**	F	58	L	NM	Atypical deformation	Degeneration	OA, disk perforation	NA

*Abbreviations*: *ADD* Anterior disk displacement, *Bi* Bilateral, *F* Female, *M* Male, *NA* Not available, *NM* Not mentioned, *L* Left, *LPM* Lateral pterygoid muscle, *OA* Osteoarthrosis/osteoarthritis, *R* Right

^a^Radiographic findings were observed through preoperative cone beam computed tomography and magnetic resonance imagings

The mean age was 45.33 ± 12.33 ranging from 23 to 67 years old, and the male-to-female ratio was 1:6.8. All patients only received conservative interventions previously, and had no history of TMJ surgery. Interventions included patient education, exercise, manual therapy, photobiomodulation, splinting, needling, and intraarticular injection. Unfortunately, not any improvements were seen, as either a standalone treatment or as an adjunct, through evaluating self-reported chewing difficulty, mastication-related pain, and bite force/ endurance outcome measures. In addition, considering the discovery of organic lesion/ severe degeneration of joint disks in radiographic examination, surgical reconstruction was thus arranged for all included patients. Each of these patients underwent the same surgical procedure following as below: TMJ interpositional arthroplasty with removal of the damaged disk and debridement of joint and TMF reconstruction. All procedures were performed by a single experienced surgeon (Prof. Gong).

Average follow-up period was 30.07 months after surgery, with a range of 6 to 95 months. Comprehensive radiographic studies were performed preoperatively; and contained wide open and closed mouth cone beam computed tomography (CBCT) as well as the oblique sagittal (wide open and closed mouth), axial (wide open and closed mouth), and coronal (closed mouth) planes of magnetic resonance imaging (MRI) scanning so that the projection angle was in line with Schüller’s position [15]. Preoperative and postoperative subjective evaluation was designed performing a visual analogue scale (VAS) scores to evaluate pain (0 ~ 10, 0 being “no pain”, 10 being “the most pain imaginable”) [16]. Preoperative and postoperative objective assessments embraced joint noise on function, deviation on opening, range of movement, and cosmesis.

### Surgical technique

**Fig. 2:**
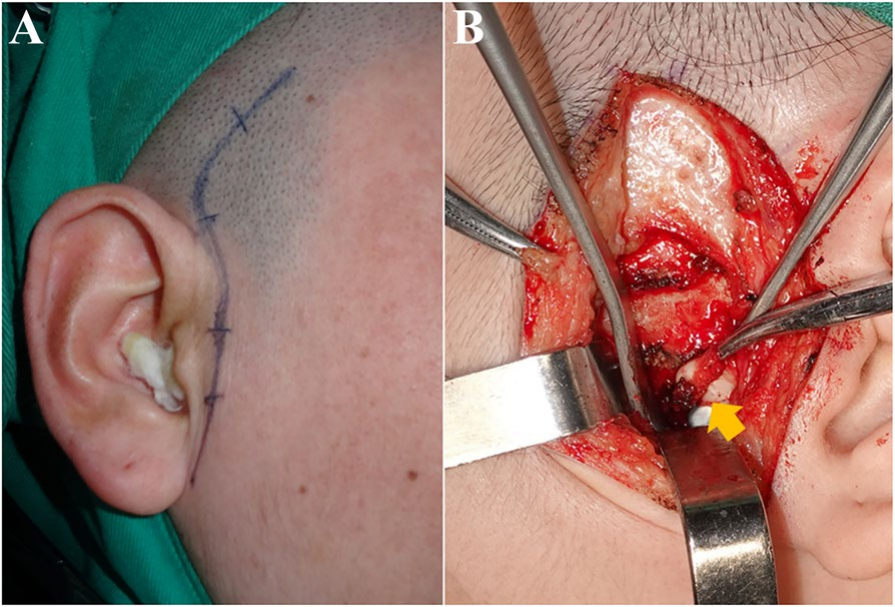
**A** Preauricular skin incision for TMJ arthroplasty with temporalis myofascial flap reconstruction. **B** Disk perforation was presented via T-shaped incision of the joint capsule (indicated by the arrow)

The TMJ was approached through a preauricular incision with a 1.5 ~ 2.0 cm extension into the temporal hairline. Local hemostatic solution (with an adrenaline: normal saline ratio of 1 mL: 200 mL) was infiltrated into the skin and underlying tissues to reduce bleeding at the site of surgery. Dissection was conducted via the superficial temporal fascia. The fascia, along with the facial nerve (mainly temporal branch), were anteriorly retracted; and the periosteum wrapped over the posterior zygomatic arch was incised. Exposure of the condylar eminence and articular fossa of TMJ was accomplished by T-shaped incision of the joint capsule. This approach only incised the superficial fasciculi of lateral ligament to minimize the injury of peripheral soft tissue **(**Fig. 2**)**. Surgical debridement of the joint, including removal of perforated or residual disk and affected soft tissue, was performed. If necessary, the erosive condylar/ articular surfaces were recontoured using a reciprocating rasp.

**Fig. 3:**
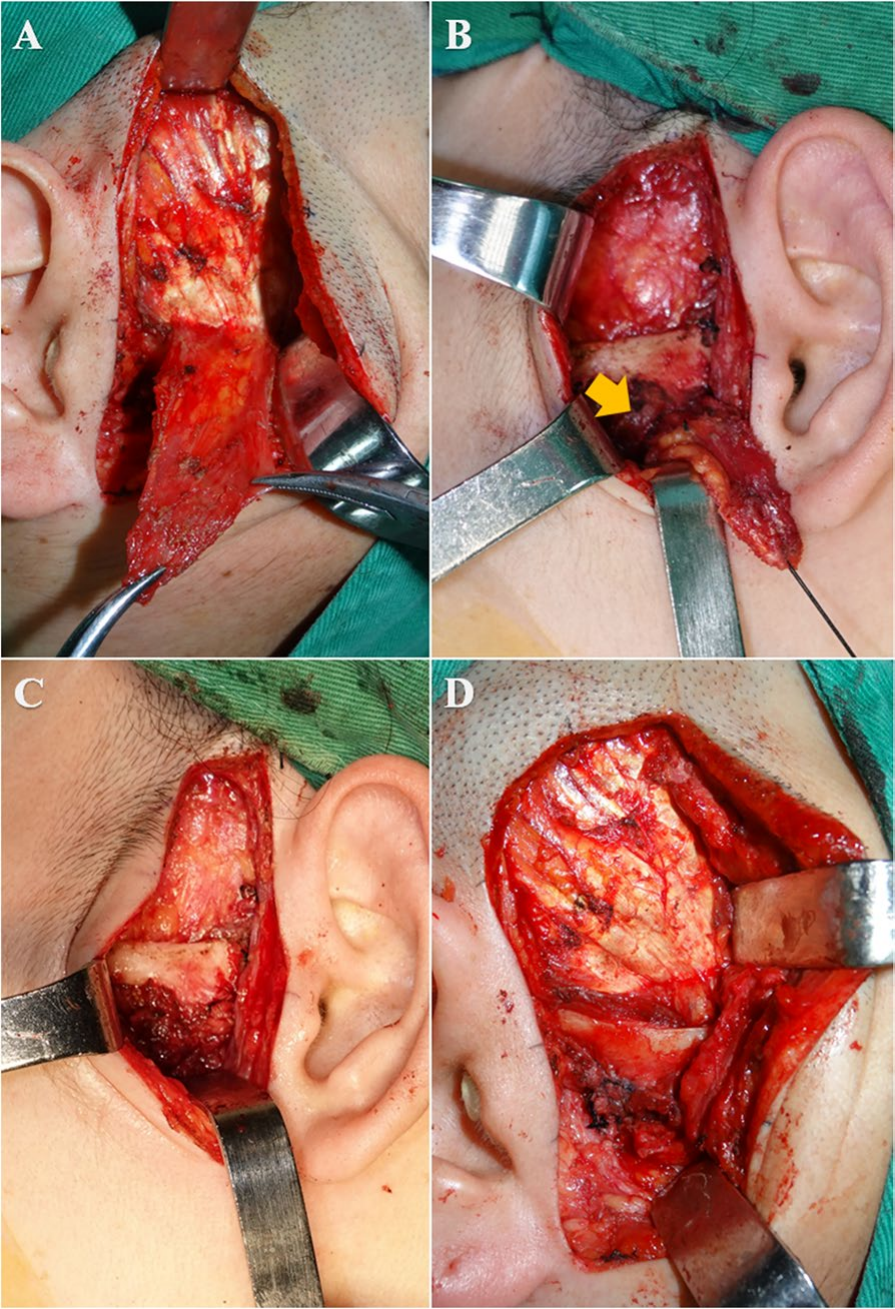
Exposure of the joint and donor region was ready for preparation and transference of the temporalis myofascial flap. **A** Pedicled temporalis myofascial flap reflected away from intramuscular fascia. **B** The flap passed through inferiorly deep surface of zygomatic arch, and was inserted into the joint cavity from the deep fasciculi of lateral ligament which was retained. **C** and **D** The interpositional flap covered the top of the condyle and sutured with joint capsule of bilaminar zone

Once the diskectomy was completed as well as the joint was debrided, the area of pedicled TMF to be proposed was outlined in methylene blue and local hemostatic infiltrated. A posteriorly oriented flap based on the deep temporal vasculature was preferably designed in order to obtain a good orientation relative to the condyle and fossa, when the flap was rotated inferiorly. Within the temporalis muscle proper, there is an intramuscular fascia that divides the more superficial portion of muscle from the deeper portion [17]. The flap was taken from the middle portion of the muscle; and dissection of the outlined flap was carried through to the intramuscular fascia, and a flap consisting of superficial temporalis muscle was created **(**Fig. 3A**)**.

Unlike conventional way of TMF transference (a zygomatic arch osteotomy described previously in some studies [18–20]), in this surgery, taking the pedicle of TMF as the center, the flap was turned over and through inferiorly deep surface of zygomatic arch and secured in the joint space with nonabsorbable 4–0 PROLENE braided polyester fiber suture (W8557, Ethicon Ltd., USA), so as to allow for atraumatic flap transposition **(**Fig. 3B-D**)**. Hemostasis might be achieved by electrocautery, and drains were generally not necessary, and pressure dressings were applied.

### Postoperative points for attention

All patients were required to have a liquid diet within one week, then gradually to have transition to soft diet and general diet. One week after surgery, all patients started their active mouth-opening excises with frequency of 3 to 5 times per day, at least 10 to 15 minutes each time. Additionally, after a week of active motion exercise, all patients initially underwent supervised physical therapy of passive range of motion exercises at least 3 days per week for 3 weeks. Subsequently, they continued daily physical therapy at home for an additional 2 to 3 weeks.

### Statistical analysis

Statistical analysis was performed using Statistical Analysis System software (version 9.1.3; SAS Institute Inc., Raleigh, North Carolina, USA). The Kolmogorov–Smirnov test as a priori statistical assessment was used to verify the normality of all data. Data conforming to the Gaussian distribution (Sig > 0.05) used for paired *t*-test. Otherwise the statistical method applied for non-normally distributed data was Wilcoxon signed-rank sum test, with *P* <  0.05 as significant difference.

## Results

### Preoperative radiographic evaluation

**Fig. 4:**
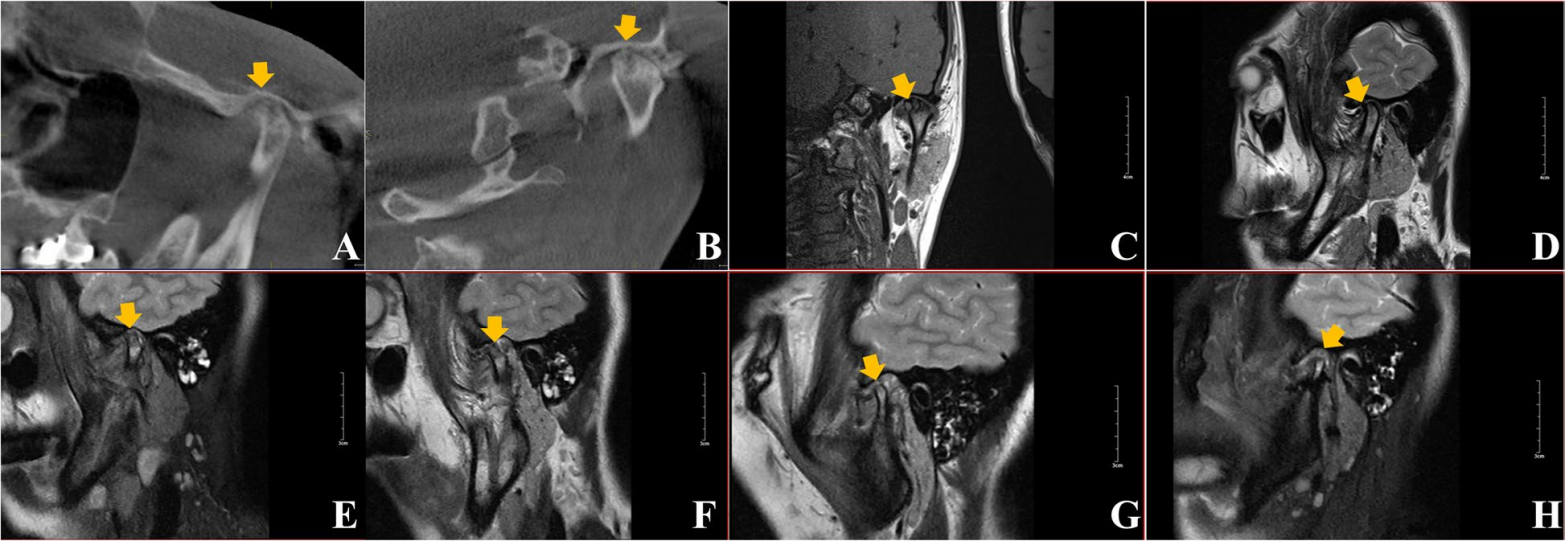
Preoperative (**A-D**) and postoperative (**E-H**) radiographic results. Fig. **A** was visualized by oblique sagittal position of cone beam computed tomography (CBCT), **B** was oblique coronal position of CBCT image; Fig. **C** was visualized by oblique coronal position of magnetic resonance imaging (MRI), **D** was oblique sagittal position of MR image. Figs. **E**, **F**, **G**, and **H** were MRI oblique sagittal slices, with the performance of animate muscle tissue or/and accompanied by adipose tissue indicated by the graft of temporalis myofascial flap in joint space

A total of 50 joints were examined; of these assessed joints, 44 showed signs of osseous degeneration, including cortical erosion, condylar flattening, and joint space alterations; 4 joints indicated irregularly abnormal morphology; and 2 joints indicated condylar head displacement (Table 1) (Fig. 4A-D).

### Pre- and post-operative subjective assessment

Preoperatively, all patients had some degree of pain in the affected joint, ranging from 4.5 to 10 (average score, 8.22 ± 1.94) according to the VAS standard (10 scores being “the most pain imaginable”). Postoperatively, this range was 0 to 9.5, and the mean pain score was 2.21 ± 2.75 (Table 2). The pain score was significantly decreased after reconstructive surgery of disk using TMF transplantation (*P* < 0.001). Fourteen patients rated their pain as “0” postoperatively; five patients complained their pain did not ameliorate after the procedure, but no patient reported an increase in pain evaluation.

**Table 2:** Preoperative and postoperative pain management

Case no.	Affected side (L/R/Bi)	Pain VAS before TMF transplantation	Pain VAS after TMF transplantation	Postoperative pain medications
**1**	Bi	10	0	None
**2**	L	10	2	Narcotic for flare-ups
**3**	Bi	6	0	None
**4**	R	10	0	Occasional NSAIDs (Paracetamol)
**5**	R	7	5	NM
**6**	L	4.5	1	Occasional NSAIDs (Paracetamol)
**7**	L	10	0	NM
**8**	L	9	7	Occasional NSAIDs (Ibuprofen)
**9**	Bi	10	3	Occasional NSAIDs (Paracetamol)
**10**	R	7	0	Occasional NSAIDs (Ibuprofen)
**11**	R	8	8	Occasional NSAIDs (Ibuprofen); Narcotic for flare-ups
**12**	Bi	10	9.5	Narcotic for flare-ups
**13**	Bi	2	2	Occasional NSAIDs (Ibuprofen)
**14**	Bi	9	9	Narcotic for flare-ups
**15**	R	10	6	Occasional NSAIDs (Paracetamol)
**16**	R	10	2.5	Occasional NSAIDs (Ibuprofen); Narcotic for flare-ups
**17**	R	10	1	Ibuprofen; Diclofenac sodium
**18**	R	8	4	NA
**19**	Bi	5	5	Occasional NSAIDs (Ibuprofen); Narcotic for flare-ups; Muscle relaxant (Cyclobenzaprine)
**20**	L	10	2.5	Occasional NSAIDs (Ibuprofen); Narcotic for flare-ups
**21**	L	8	6.5	Narcotic for flare-ups
**22**	R	9	0	NM
**23**	Bi	8	2	None
**24**	L	9	1	None
**25**	R	10	1	Occasional NSAIDs (Ibuprofen)
**26**	Bi	7	1	None
**27**	Bi	8	0	Occasional NSAIDs (Ibuprofen)
**28**	L	8	0	None
**29**	R	9	0	Narcotic for flare-ups
**30**	L	7	2	Narcotic for flare-ups
**31**	R	10	0	Occasional NSAIDs (Ibuprofen)
**32**	R	10	0	Occasional NSAIDs (Ibuprofen)
**33**	R	6	0	None
**34**	L	9	1	None
**35**	L	8	2	Occasional NSAIDs (Paracetamol)
**36**	L	5	1	Occasional NSAIDs (Paracetamol)
**37**	L	10	0	Occasional NSAIDs (Paracetamol)
**38**	Bi	8	0	None
**39**	L	6	1	NA
Average VAS score		8.22 ± 1.94	2.21 ± 2.75	***P*** value* < 0.001

*Abbreviations*: *Bi* Bilateral, *NA* Not available, *NM* Not mentioned, *L* Left, *NSAIDs* Nonsteroidal anti-inflammatory drugs, *R* Right, *TMF* Temporalis myofascial flap, *VAS* Visual analogue scale

*P < 0.05 was considered as significant

### Pre- and post-operative objective assessment

To assess the range of motion, the maximum mouth opening (MMO) was measured before and after operation (Table 3). The average preoperative MMO value was 29.15 ± 6.98 mm (ranging from 12 to 39 mm), and the average postoperative MMO value was 35.77 ± 4.47 mm (ranging from 25 to 42 mm). 32 of 39 patients showed an increase in MMO postoperatively, three patients had no change in MMO, and 4 had an average decrease of 2.25 mm. The mean increase of MMO value after disk reconstruction with the TMF was 7.74 mm. There was a significant increase in MMO postoperatively compared with the preoperative average (*P* < 0.001).

**Table 3:** Preoperative and postoperative assessment of function

Case no.	Affected side (L/R/Bi)	Initial MIO (mm)	Postsurgical MIO (mm)	***P*** value*
**1**	Bi	32	34	*P* < 0.001
**2**	L	27	40	
**3**	Bi	12	29	
**4**	R	20	32	
**5**	R	23	39	
**6**	L	18	35	
**7**	L	13	28	
**8**	L	30	40	
**9**	Bi	32	34	
**10**	R	33	38	
**11**	R	36	35	
**12**	Bi	20	41	
**13**	Bi	30	30	
**14**	Bi	38	34	
**15**	R	28	31	
**16**	R	32	35	
**17**	R	32	35	
**18**	R	34	35	
**19**	Bi	32	31	
**20**	L	28	40	
**21**	L	33	45	
**22**	R	34	40	
**23**	Bi	39	40	
**24**	L	35	32	
**25**	R	18	30	
**26**	Bi	24	40	
**27**	Bi	30	42	
**28**	L	36	38	
**29**	R	35	39	
**30**	L	38	38	
**31**	R	18	35	
**32**	R	29	31	
**33**	R	27	40	
**34**	L	35	36	
**35**	L	33	38	
**36**	L	28	32	
**37**	L	37	41	
**38**	Bi	25	25	
**39**	L	33	37	
Average MMO (mm)		29.15 ± 6.98	35.77 ± 4.47	

*Abbreviations*: *Bi* Bilateral, *L* Left, *MIO* Maximal interincisal opening, *R* Right

*P < 0.05 was considered as significant

## Discussion

As the only hinge type synovial joint with articular disk, TMJ is featured with intricate structural components to behave in a unique way of bilateral movements namely rotational movement (depression/elevation) and translational movement (lateral deviation and retraction/protrusion). The articular disk is an intrinsic structure of slim and oblong plate that plays crucial role during chewing, as it sustains an enormous amount of pressure to prevent the articular surfaces of bones from coming into contact with each other. For this reason, the disk is a fragile component of the joint especially susceptible to abnormal jaw movement. Patients with serious disk dysfunction or particularly with interpositional TMJ graft failure often pose a challenge for reconstruction and management. These patients frequently have difficulty in maintaining a normal diet resulting from significant pain and limitation of movement.

Autogenous-tissue flap transplantation is still an important method for reconstruction of articular disk after its resection. By reviewing the available literatures, the TMF could be considered as an optimal option compared with abdominal dermis-fat graft, auricle cartilage, *etc* [21, 22]. TMF is a sort of versatile tool in reconstructing disk, which is possible to use the temporal fascia flap pedicled to the middle temporal vessels, or muscular temporalis flap pedicled to the deep temporal vessels [17, 23]. With regard to TMJ disk replacement, the TMF can meet the physiological function of a disk supplying as autologous tissue. Because this flap is attached to the mandibular condyloid process, it is theoretically pulled forward and down as the condyle translates, therefore simulating the dynamic function of the disk.

Many clinicians have advocated the placement of an interpositional material in the joint after diskectomy to avoid crepitus, degenerative changes, pain, ankylosis, occlusional alterations, and limited movement [5, 13, 14]. As a result, the application of TMF in joint surgery was reported by the literature, including TMJ ankylosis, tumour resection, traumatic defect, congenital malformation, joint degeneration, previous failed interpositional TMJ graft procedures, *etc*, as an implant or disk replacement material [19, 24–26]. In this study, a group of thirty-nine cases (fifty sides) was treated with TMF transplantation to reconstruct the articular disk for late disk displacement without reduction, disk perforation, which agreed with the indication of TMF for arthroplasty described in literatures. Our surgical technique utilized the flap that was turned over and through inferiorly deep surface of zygomatic arch and secured in the joint space, by contrast with the traditional method of TMF transference through a zygomatic arch osteotomy [18–20]. Accordingly, this surgical method did not give rise to additional damage to zygomatic arch that simplified the procedure and caused no potential bulging deformity of zygomatic arch postoperatively. Another modification regarding the design of operative approach for joint cavity was a T-shaped incision on joint capsule. It would gain a good protection of the normal deep fasciculi of lateral ligament to a great extent, thus better stabilizing the joint after closure. The patients were followed up for 6 to 95 months, with an average period of 30.07 months after surgery. Postoperative MRI results indicated that the TMF grafts in joint space were animate muscle tissue or/and accompanied by adipose tissue, which showed this flap was capable to restore joint function and preserve its vitality after transplantation (Fig. 4E-H).

Because our feasibility study contained a relatively small sample size, our findings should be verified by studies involving larger sample sizes. Furthermore, another limitation of the present investigation include the lacking of unilateral and bilateral matching, the potential association between these parameters and different TMJ sides should be clarified. In addition, the sample size of men was smaller than that of women, reflecting the lower prevalence of TMD in men [27]. Studies with sufficient male samples are needed to avoid selective bias and better understand the effectiveness of applied new surgical technique. Randomized controlled trial (modified approach versus conventional approach) is needed to further confirm our findings based on a reliable sample size in the future.

## Conclusion

The temporalis myofascial flap is an autogenous origin that has the advantages of close proximity to the temporomandibular joint, minimal surgical morbidity, and successful clinical results. Attachment to the condyloid process is guaranteed, providing movement of the flap during function, simulating physiologic function of the articular disk. More importantly, these findings offer significant values in TMJ reconstruction with a modified surgical technique that gains atraumatic zygomatic arch as well as keeps extracapsular lateral ligament’s deep fasciculi from unnecessary harm in order that best stability can be reached postoperatively. This method is feasible, but its long-term efficacy and related issues need to be further studied.

## Data Availability

The analyzed data sets generated during the study are available from the corresponding author on reasonable request.
